# Thermal Conductivity Performance of Polypropylene Composites Filled with Polydopamine-Functionalized Hexagonal Boron Nitride

**DOI:** 10.1371/journal.pone.0170523

**Published:** 2017-01-20

**Authors:** Lin Chen, Hong-Fei Xu, Shao-Jian He, Yi-Hang Du, Nan-Jie Yu, Xiao-Ze Du, Jun Lin, Sergei Nazarenko

**Affiliations:** 1 Key Laboratory of Condition Monitoring and Control for Power Plant Equipment of Ministry of Education, North China Electric Power University, Beijing, China; 2 School of Renewable Energy, North China Electric Power University, Beijing, China; 3 School of Polymers and High Performance Materials, The University of Southern Mississippi, Hattiesburg, Mississippi, United States of America; Institute of Materials Science, GERMANY

## Abstract

Mussel-inspired approach was attempted to non-covalently functionalize the surfaces of boron nitride (BN) with self-polymerized dopamine coatings in order to reduce the interfacial thermal barrier and enhance the thermal conductivity of BN-containing composites. Compared to the polypropylene (PP) composites filled with pristine BN at the same filler content, thermal conductivity was much higher for those filled with both functionalized BN (f-BN) and maleic anhydride grafted PP (PP-g-ma) due to the improved filler dispersion and better interfacial filler-matrix compatibility, which facilitated the development of more thermal paths. Theoretical models were also applied to predict the composite thermal conductivity in which the Nielsen model was found to fit well with the experimental results, and the estimated effective aspect ratio of fillers well corresponded to the degree of filler aggregation as observed in the morphological study.

## Introduction

For heat transfer related applications such as anti-corrosion heat exchanger [1, 2], thermal interface materials [3–5] and heat sinks in electronic devices [6, 7], thermally conductive fillers have been employed to improve the thermal conductivity (TC) of polymeric materials [8, 9]. Among them, boron nitride (BN) is one of the most promising ones because it is both thermally conductive and electrically insulative [7, 10–14]. So far, BN has been used in the composites based on various matrices including rubber [15, 16], silicone gel [17], epoxy [18], polyvinyl alcohol (PVA) [13], polyimide (PI) [10], liquid crystalline polyester (LCP) [19], polybenzoxazine [20], polysiloxane[21], polyethylene (PE) [22] and polypropylene (PP) [23].

The transport of heat in non-metals has been well discussed in literature. According to Berman’s work [20, 24], the thermal resistance (TR) is mainly caused by phonon scattering processes, including phonon-phonon scattering, boundary scattering and defect or impurity scattering. In composite materials, phonon scattering is mainly due to the existence of an interfacial thermal barrier, which results from the acoustic mismatch or the damage of surface layer between the filler and polymer matrix [20, 25]. In order to lower the TR or enhance the TC of the composite, measures should be taken to reduce the interfacial thermal barrier, which is closely related to the filler dispersion and the filler-matrix interaction.

For BN-containing composites, modification of BN particles has been investigated to enhance the filler dispersion and filler-matrix contact [18, 23, 26–31]. Huang et al. prepared polyhedral oligosilsesquioxane-modified boron nitride nanotubes (BNNTs) via silane-coupling hydroxylated BNNTs, which were then applied in the fabrication of epoxy nanocomposites [26]. The TC of the composites with 30 wt% filler was 13.6 times higher than the pristine epoxy. Muratov et al. [23] used 3-amino-propyl-3-ethoxysilane (APTES) to treat hexagonal BN (h-BN) with hydroxyl surface groups that were introduced by different oxidation processes. They found that the TC of PP composites filled with surface-modified h-BN was up to 2 times as compared with the composites filled with pristine h-BN. In the study by Yu et al. [18], BN nanoplatelets were first covalently functionalized with hyperbranched aromatic polyamide (HBP). The modified BN nanoplatelets were then incorporated into epoxy to form the composites, which exhibited larger TC improvement as compared to that filled with pristine BN nanoplatelets. Due to the chemical inertness of BN, an extra “activation” step is always needed to introduce functional groups to the surfaces of BN before the further chemical reaction. This makes the covalent functionalization process relatively tedious. Therefore, non-covalent functionalization of BN was also attempted in the past. Yu et al. utilized the Lewis acid-base complexations between the electron-rich amine groups of octadecylamine (ODA) and the electron-deficient boron atoms to modify the BN nanoplatelets surface with ODA [18], which also showed better TC enhancement than the pristine BN when filled in the epoxy composites. Realizing the π-conjugation nature on the BN surfaces, conjugated molecules including catechin [27], polyaniline [29] and poly(p-phenylene-ethynylene)s [31] were employed to achieve the non-covalent functionalization of BN with the aid of strong π-π interaction. The addition of such non-covalently modified BN also resulted in the better filler dispersion and higher composite TC [27].

Recently, mussel-inspired approach has been widely employed to modify various materials with self-polymerized dopamine coatings, which was found to be able to adhere to almost all kinds of substrates [32–35]. In this work, we adopted such approach to functionalize BN surfaces with a thin layer of polydopamine coatings. In addition, the conjugated feature of polydopamine is also expected to enable strong π-π interaction with BN. Both pristine and modified BN were incorporated into PP matrix with the aim of TC enhancement. In the third series of PP composites, maleic anhydride grafted PP (PP-g-ma) was also employed as the compatibilizer, which may react with catechol and/or amine groups in polydopamine coatings of BN and improve the dispersion of BN particles in the composites. TC properties of these three series of PP composites were compared and correlated with the filler dispersion and the interfacial interaction between the filler and polymer matrix.

## Experimental

### Materials

Polypropylene (PP, Moplen RP344RK) with molecular weight of ~100,000 Dalton and density of 0.9 g/cm^3^, was manufactured by PolyMirae, Korea. Hexagonal boron nitride (h-BN) with diameters of 5~10 μm and purity of 99.0% was supplied by Eno Material, China. Maleic anhydride grafted PP (PP-g-ma) with molecular weight of ~9100 Dalton and maleic anhydride of 8~10 wt% was purchased from Sigma-Aldrich, USA. Dopamine hydrochloride (99%) and tris(hydroxymethyl)-aminomethane hydrochloride (Tris-HCl) was supplied by Aladdin, China. All materials were used as received.

### Modification of h-BN

The h-BN was ultrasonically dispersed in de-ionized (DI) water (25g/625mL) for 30 mins, before the addition of dopamine (1g/L) and tris-HCl (10 mmol/L). The pH value of the solution was adjusted to 8.5 by using NaOH solution (0.1 mol/L). After being stirred for 6 h, the mixture was centrifuged and washed by DI water for 10 times, and then was dried at 40°C in vacuum. The surface-modified h-BN is denoted as f-BN.

### Preparation of composites

Three series of BN-filled PP composites were prepared. The first series of PP composites was filled with pristine h-BN, which is referred to as “PP/BN” composites. The second series PP composites was filled with f-BN and is referred to as “PP/f-BN” composites. For the third series of PP composites, 2.5 wt% of PP-g-ma was added to PP matrix in addition to the f-BN fillers. This series is referred to as “PP/PP-g-ma/f-BN” composites. For all the three series of PP composites, the filler content varied from 5 wt% to 25 wt%, where the filler referred to either BN or f-BN.

PP, PP-g-ma and BN fillers were dried at 80°C for 12 h before being mixed and extruded at 190°C in a twin-screw extruder. The extruded composites were hot pressed at 200°C and 16 MPa to obtain the films with various thickness for characterization.

### Characterization and measurements

Thermogravimetry analysis (TGA) was carried out on a thermal analyzer (Q500, TA, USA) under nitrogen atmosphere at a heating rate of 20°C/min. Fourier transform infrared spectra (FTIR) were recorded on a NICOLET iS10 system (Thermo Fisher Scientific, USA) in the range of 4000~400 cm^-1^. The filler distribution in the composites was observed by ultra-high resolution scanning electron microscope (SEM, SU8010, Hitachi, Japan). The samples were freeze-fractured in liquid nitrogen and sputtered with a conductive layer of gold under vacuum. A telescopic goniometer (OCA15 EC, Dataphysics, Germany) with 5 μl water droplets was used to measure the static water contact angles of the h-BN and f-BN particles by sessile drop method at ambient temperature. For each sample, at least eight measurements were performed on different surface locations and the average values were reported. The composite TC is calculated by Eq (1),

k=α⋅ρ⋅c (1)

where *α*, *ρ* and *c* are the thermal diffusivity, density and heat capacity of the composite, respectively. The density was determined by a density tester (MH-300A, Qunlong, China) at 30°C. The thermal diffusivity was measured by laser flash method (LFA 447, Netzsch, Germany) at 30°C. The heat capacity was measured by using a differential scanning calorimetry analyzer (DSC 404 C, Netzsch, Germany). For heat capacity measurement, all samples were crimped in non-hermetic aluminum pans with lids. The weight of the samples was in the range of 10 ~ 15 mg. Sapphire was used as the reference to calibrate the instrument. The samples were equilibrated for 10 min at 20°C before being heated at a rate of 5°C/min to 100°C. All heat capacity values used for thermal conductivity calculated were determined at 30°C.

## Results and Discussion

**Fig 1:**
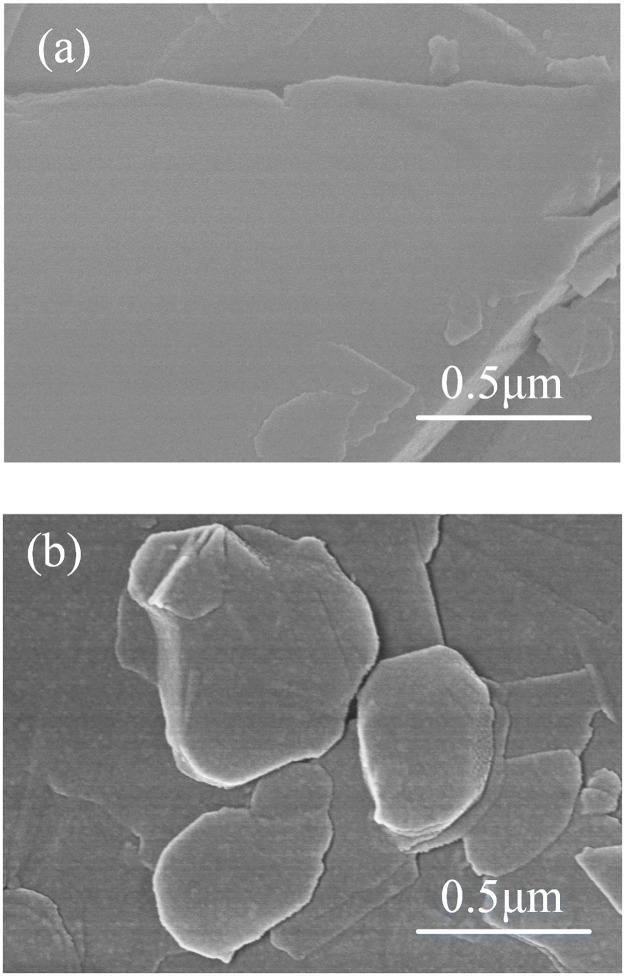
SEM images of (a) pristine h-BN and (b) f-BN.

SEM analysis was first performed on pristine h-BN and f-BN to study the coating of h-BN with self-polymerized dopamine. As shown in Fig 1, the surface of f-BN becomes much rougher compared to the pristine h-BN. This suggests that the polydopamine layers have been successfully deposited onto the surface of h-BN.

**Fig 2:**
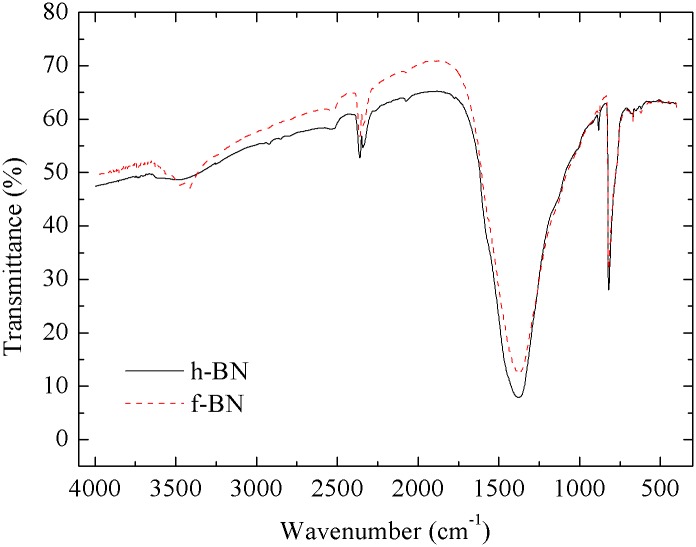
Fourier transform infrared spectroscopy (FTIR) of h-BN and f-BN.

The existence of polydopamine layers in f-BN was also confirmed by the FTIR results shown in Fig 2. Compared to that of h-BN, the broad absorption bands at 3200–3700 cm^-1^ become much stronger for f-BN, which may be due to the stretching vibration of -OH and–NH groups of polydopamine [36]. In addition, the average contact angle of f-BN was measured to be 57° (standard deviation *σ* = 0.52), much lower than that of pristine h-BN of 108° (standard deviation *σ* = 1.26), indicating much increased hydrophilicity of the BN particles after surface modification with polydopamine layers.

**Fig 3:**
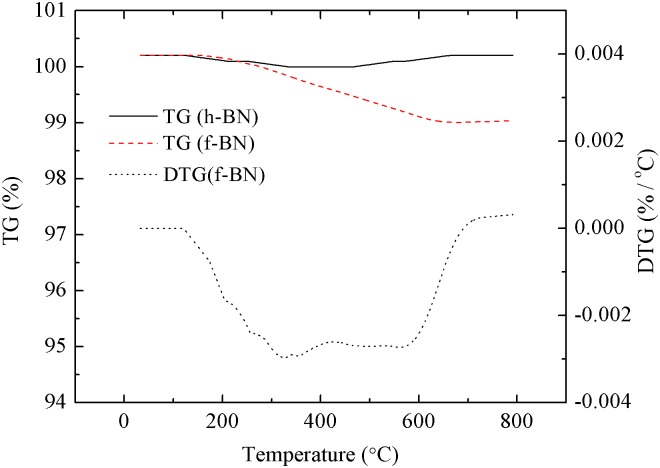
Weight loss curves of h-BN and f-BN.

As shown in Fig 3, the pristine h-BN particles demonstrate high thermal stability as they experience insignificant weight loss up to ~800°C. As for the f-BN, the weight loss starting from ~300°C may be related to the decomposition of polydopamine deposited on the surface of f-BN. Under N_2_ atmosphere, there is around 50% weight loss of polydopamine at ~800°C [37]. Therefore, the amount of deposited polydopamine could be estimated to be around 2 wt%, close to the value (1.8 wt%) determined from the elemental analysis experiments.

**Fig 4:**
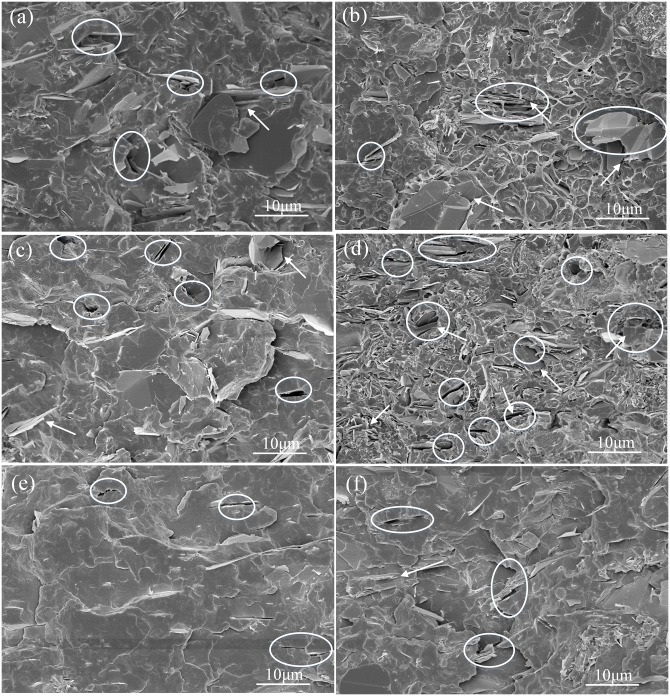
SEM images of PP composites. (a) PP/BN composites with 10 wt% pristine h-BN; (b) PP/BN composites with 20 wt% pristine h-BN; (c) PP/f-BN composites with 10 wt% f-BN; (d) PP/f-BN composites with 20 wt% f-BN; (e) PP/PP-g-ma/f-BN composites with 10 wt% f-BN; (f) PP/PP-g-ma/f-BN composites with 20 wt% f-BN.

The filler dispersion in the polymer matrix for three series of composites was investigated by SEM. As shown in Fig 4a and 4b, the BN fillers are dispersed quite well in PP, with small portion of voids (pointed by circles) and agglomeration of the filler particles (pointed by arrows). However, when the BN filler was modified with polydopamine, the amount of voids in the PP/f-BN composites becomes larger and the filler agglomeration becomes more severe, even though the filler dispersion is still relatively homogeneous (Fig 4c and 4d). Such phenomenon may be correlated with the increased hydrophilicity of f-BN fillers that leads to the higher incompatibility with the hydrophobic PP matrix. With the addition of PP-g-ma, the interfacial compatibility between the filler and the matrix is much improved as much less voids and better fill dispersion can be observed in the composites (Fig 4e and 4f). Such enhancement may result from the compatibilization effect of PP-g-ma, which preferentially resides at the interface of PP and f-BN and improves interfacial adhesion through the formation of chemical bonding between the anhydride groups of PP-g-ma and the hydroxyl and amine groups of polydopamine layers on the surface of f-BN. As will be discussed in the sections below, the difference in the filler dispersion and interfacial adhesion between the filler and the matrix will significantly affect the composite TCs.

**Fig 5:**
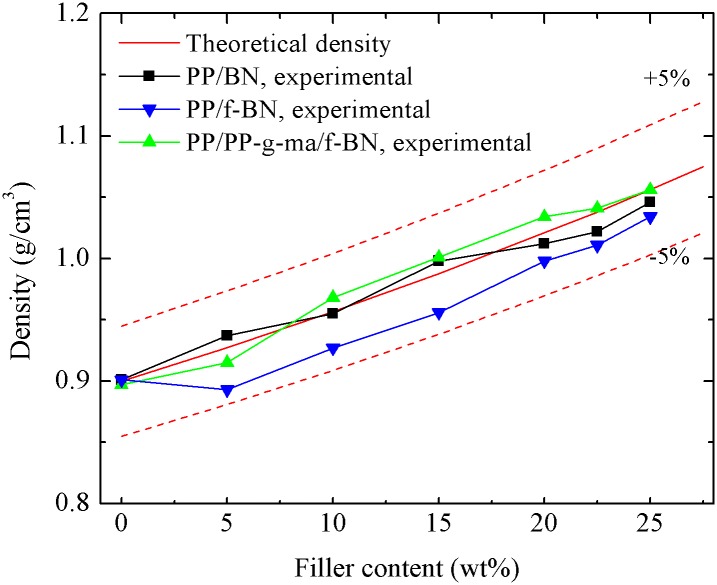
Densities of different series of composites.

The theoretical density of PP/BN composites is calculated with the density of h-BN taken as 2.25 g/cm^3^ [20]. As for PP/PP-g-ma/f-BN and PP/f-BN composites, on the one hand, there is only 2.5wt% PP-g-ma added to the PP matrix; on the other hand, the amount of polydopamine in f-BN is relatively small. Therefore, the theoretical density of PP/BN composites is also taken as that of PP/PP-g-ma/f-BN and PP/f-BN composites. Fig 5 shows the measured density results of BN-filled composites as a function of filler content. Generally, the experimental results agree well with the theoretical one, i.e., the deviations are within ±5%. Such results suggest that the interfacial compatibility between the filler and the polymer matrix is relatively good for these composites, which is also confirmed by the SEM results discussed earlier. However, it should be noted that the experimental density of PP/f-BN composites is consistently lower than the theoretical values. This could indicate slightly poor interfacial adhesion between the PP matrix and f-BN, which correlates with the existence of slightly more voids in PP/f-BN composites shown in the SEM studies (Fig 4c and 4d).

**Fig 6:**
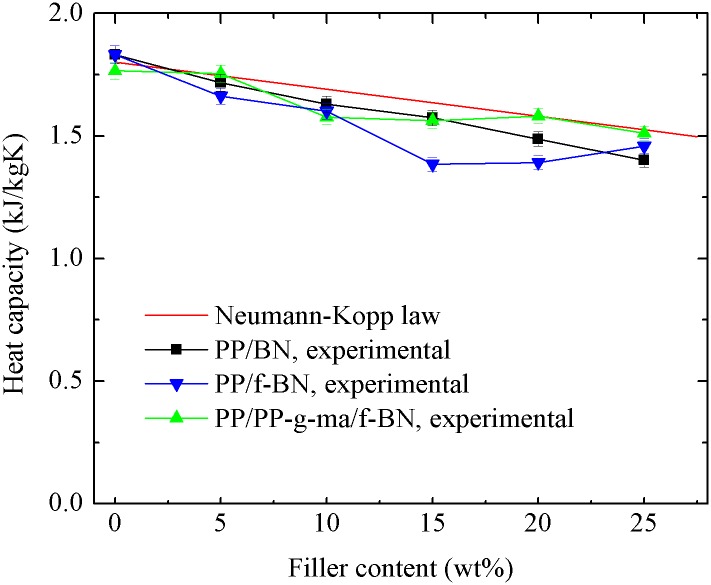
Heat capacities of different series of composites.

Fig 6 shows the heat capacity results of BN-filled composites. Since the empirical Neumann-Kopp law can give a reasonable estimate of heat capacity for a mixed material [38], the heat capacity of composites, *c*_*p*,*c*_, is also calculated by

$$c_{p}{}_{,c}=c_{p}{}_{,BN}\cdot \varphi_{BN}+c_{p}{}_{,PP}\cdot (1-\varphi_{BN})$$ (2)

where *φ*_*BN*_ is the weight percent of BN filler, *c*_*p*,*BN*_ and *c*_*p*,*PP*_ are heat capacities of BN and PP, with the values of 0.7 and 1.8 kJ/kgK, respectively [39]. It could be found that the experimental heat capacities of PP/BN and PP/PP-g-ma/f-BN composites generally agree well with the calculation results. However, for the PP/f-BN composites, the differences between the experimental and calculation results are somewhat larger than those of the other two series of composites. This may be attributed to the more voids in the PP/f-BN composites as shown in the SEM images, since the heat capacity of air is about 1 kJ/kgK, much smaller than that of PP.

**Fig 7:**
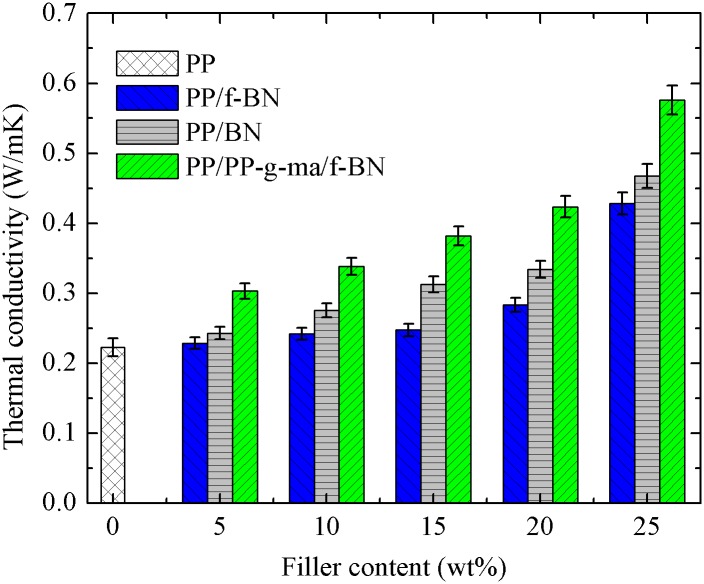
Thermal conductivity of the three series of polymer composites as a function of filler loading.

Fig 7 shows the TC results of the prepared composites. For each series of these composites, the TC increases with the increase of the filler content and begins to increase much more rapidly when the filler content is higher than ca. 20 wt% (9.3 vol%). For PP/BN composites, the TC achieves 0.47 W/mK when the filler content increases to 25 wt% (12 vol%), an enhancement of 2.14 times as compared with that of neat PP (0.22 W/mK). In a previous study by W. Cheewawuttipong et al. [40], the TC of the PP/BN composites with 15 vol% BN was shown to be ~0.60 W/mK. Assuming the linear increase of TC with the filler content when the filler content is below 15 vol%, the TC of the PP/BN composites with 12 vol% BN is estimated to be ~0.48 W/mK, which is very close to our results. With the addition of PP-g-ma and f-BN, PP/PP-g-ma/f-BN composites exhibit larger TC increase, with the highest TC of 0.58 W/mK achieved at the filler content of 25 wt%, an enhancement of 2.64 times compared to that of PP. In comparison, for PP/f-BN composites, the TC improvement with the filler incorporation is not as significant, with the highest TC reaching 0.43 W/mK, only 1.95-time enhancement. For the same filler content, the TCs of the composites always follow the order of PP/PP-g-ma/f-BN > PP/BN > PP/f-BN.

**Fig 8:**
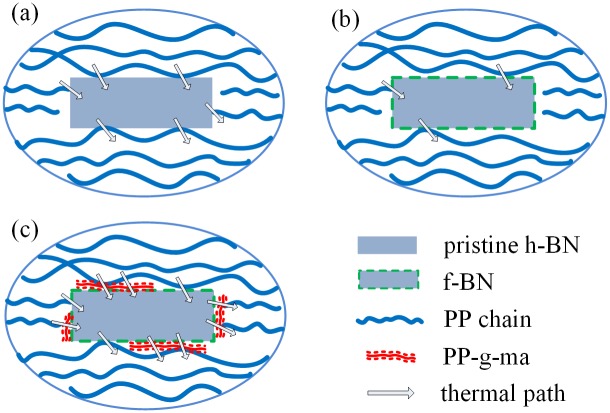
Schematic diagram of different series of composites. (a) PP/BN; (b) PP/f-BN; (c) PP/PP-g-ma/f-BN.

To explain the difference in TCs of these three series of PP composites, the schematic diagrams of thermal conduction in the composites are shown in Fig 8. As described in literature, heat transfer in polymer composites can be explained by the propagation of phonons or flow of lattice vibrational energy [20, 24]. Therefore, thermal paths are more likely to be formed at the places where the fillers are very close to the PP chains so that the thermal resistance at the filler-matrix interfaces can be minimized, as indicated in Fig 8a. For the PP/f-BN composites, the coating of polydopamine layers increases the polarity and hydrophilicity of fillers, which will be less compatible with the non-polar and hydrophobic PP matrix. The resulting void appearance shown in SEM images and the sample density lower than the theoretical prediction both confirm the worsening compatibility between the filler and the matrix for the PP/f-BN composites. As a result, some thermal paths are interrupted as indicated in Fig 8b, and the TCs of the PP/f-BN composites are lower than those of the PP/BN composites when the filler content is the same. After PP-g-ma of relatively low molecular weight is added to the composite, the voids between the PP matrix and the fillers can be filled because PP-g-ma acts as the compatibilizer which could form covalent bonding with polydopamine on the filler surfaces. Therefore, phonon scattering at the filler-matrix interface is greatly suppressed and additional thermal paths can be formed via these covalent bonds, resulting in the much enhanced heat conduction, as indicated in Fig 8c. It is also worth pointing out that the introduction of PP-g-ma alone also helps improve the composite TCs as the TCs of PP/PP-g-ma/BN composites are always higher than that of PP/BN composites at the same filler content, as shown in S1 Fig. However, the TC enhancement effect due to the polydopamine coating is still quite significant as the PP/PP-g-ma/f-BN composites always exhibit higher TCs than the corresponding PP/PP-g-ma/BN composites.

The TC behaviors of polymer composites can also be investigated quantitatively by using theoretical models [41–44]. In this work, three typical models are employed to estimate the TCs of the composites. The first model applied here is the classical Maxwell-Eucken model [45] and the composite TC is calculated by Eq (3).

$$\frac{k_{c}}{k_{m}}=\frac{2k_{m}+k_{f}+2\phi_{f}(k_{f}-k_{m})}{2k_{m}+k_{f}-\phi_{f}(k_{f}-k_{m})}$$ (3)

where *ϕ*_*f*_ is the filler volume content, *k*_*c*_, *k*_*m*_ and *k*_*f*_ are the TCs of the composite, matrix and filler, respectively. The TCs of BN and PP are set to 300 W/mK [44] and 0.22 W/mK for calculation.

**Fig 9:**
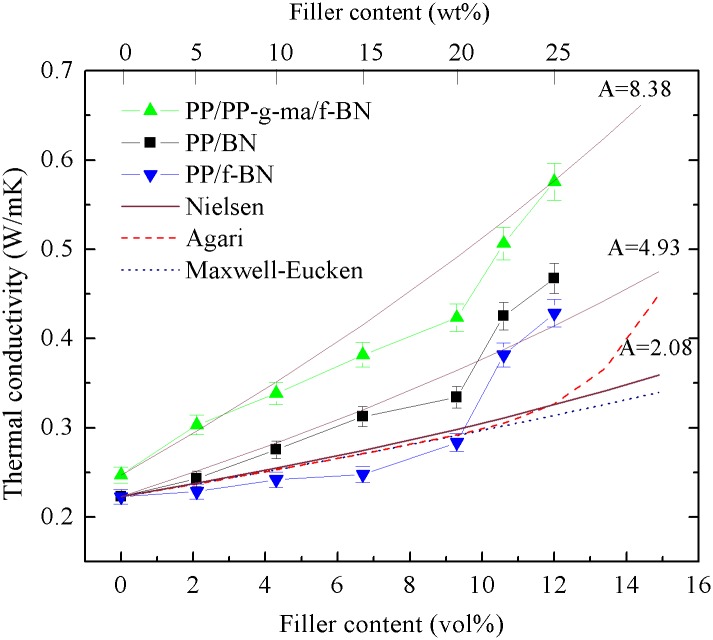
Comparison between experimental and theoretical results for the thermal conductivity of polymer composites.

As can be seen from Fig 9, the TC results calculated based on the Maxwell-Eucken model are close to those of the PP/f-BN composites and lower than the other two series of composites. As reported in literature, the Maxwell-Eucken model underestimated the TCs of the composites containing non-spherical fillers, attributed to the fact that the Maxwell-Eucken model was developed for composites containing diluted spherical fillers [45]. Therefore, the relatively good fit between the model prediction and experimental results of PP/f-BN composites may result from the following two factors: the existence of voids in the composites could contribute to the TC reduction of the composites; the agglomeration of filler particles reduces the filler aspect ratio, as observed in SEM images shown in Fig 4c and 4d.

In a model proposed by Agari [45], the Maxwell-Eucken model is modified by combining with a thermal conduction model which accounts for the effect of conductive chain formation in composites. The composite TC is calculated by

$$k_{c}=\frac{2k_{m}+k_{f}+2\phi_{af}(k_{f}-k_{m})}{2k_{m}+k_{f}-\phi_{af}(k_{f}-k_{m})}k_{m}\text{+}\phi_{f}\cdot \phi_{f}{}^{\phi_{f}{}^{-2/3}}\cdot C^{2}\cdot k_{f}$$ (4-1)

where *ϕ*_*af*_ is the volume content of particles not contributing to formation of conductive chains [45], which equals

$$\phi_{af}=(1-\phi_{f}{}^{\phi_{f}{}^{-2/3}})\cdot \phi_{f}$$ (4-2)

The relationship between *ϕ*_*f*_ and *C* is [45, 46]

$$\phi_{f}=3C^{2}-2C^{3}$$ (4-3)

The TC results calculated by Agari model are very close to those by the Maxwell- Eucken model when the filler content is lower than 9.3 vol% (20 wt%). However, when the filler content exceeds ~10 vol%, the predicted TC by Agari model increase much more rapidly, indicating that conductive chains are formed in the composite (i.e, percolation occurs) [45]. Such behavior is quite similar to that of the experimental results. As shown in Fig 9, the composite TCs start increasing much faster when the filler content is above ~10 vol%, suggesting the appearance of percolation-like behavior of fillers within the polymer matrix. According to the classical percolation theory [47], when the filler volume fraction *φ*_*f*_ reaches a critical value *φ*_*f*,*C*_ (percolation threshold), a conductive network is formed and the composite conductivity increases much more quickly with the increase of the filler content. Mathematically, the percolation can be judged by solving the parameter *t* in the following equation [48].

$$\text{log}\left(k-k_{C}\right)=a+t\text{log}(\phi_{f}-\phi_{f,c})$$ (5)

where *k*_*C*_ is the composite TC when the filler content equals *φ*_*f*,*C*_. The parameter of *t* should be close to 2 for three-dimensional randomly distributed objects when the percolation happens. Based on the experimental results obtained in this work, *φ*_*f*,*C*_ is assumed to be 9.3 vol%. By plotting log(*k*−*k*_*C*_) against log(*φ*_*f*_ −*φ*_*f*,*C*_), *t* could be calculated to be about 0.52, 0.53 and 0.83 for PP/f-BN, PP/BN and PP/PP-g-ma/f-BN composites, respectively. Obviously, the results for parameter *t* for the composites prepared in the current work are much smaller than the theoretical value of 2 [47], indicating that the observed relatively rapid increase of TC is not completely governed by the percolation concept.

By considering geometry and packing efficiency of fillers, the Nielsen model [49, 50] is utilized to predict the composite TCs according to the following equations:

$$\frac{k_{c}}{k_{m}}=\frac{1+AB\phi_{f}}{1-B\Psi \phi_{f}}$$ (6-1)

$$B=\frac{k_{f}/k_{m}-1}{k_{f}/k_{m}+A}$$ (6-2)

$$\Psi ≅1+\frac{1-\phi_{m}}{\phi_{m}^{2}}\phi_{f}$$ (6-3)

where *ϕ*_*m*_ is the maximum packing fraction of the dispersed fillers, and *A* is a parameter depending on the shape and orientation of fillers. The *ϕ*_*m*_ is chosen as 0.52 in this work, since the BN fillers are randomly oriented in the composites [50]. As shown in Fig 9, the calculated results by the Nielsen model fit quite well with the experimental ones when the value of parameter *A* was chosen as 8.38, 4.93 and 2.08, respectively, which corresponds to randomly oriented fillers with aspect ratios of 15, 10 and 4, respectively, according to Table 1 of Ref [50]. For the PP/BN composites, the effective aspect ratio of the fillers is slightly lower than that of the BN platelets, which is about 12 ~ 15. This is mainly because there is modest aggregation of BN fillers in the composites, as observed in SEM study shown in Fig 4a and 4b. As for the PP/f-BN composites, the poor compatibility between the polar f-BN and the nonpolar PP matrix leads to relatively non-uniform dispersion of the fillers. Furthermore, due to the π-π interaction between the surface-coated polydopamine layers, the f-BN particles trend to aggregate more easily than the pristine BN. Consequently, the effective aspect ratio of f-BN fillers in the PP/f-BN composites decreases to a much smaller value, which can be confirmed by the SEM observation in Fig 4c and 4d. In comparison, for the PP/PP-g-ma/f-BN composites, the effective aspect ratio of fillers predicted by the Nielsen model is very close to that of the BN platelets, indicating that the extent of filler aggregation in the composites is quite insignificant if there is any. Such results may be related to the improved compatibility between the fillers and the polymer matrix due to the strong interaction between PP-g-ma and f-BN. SEM characterization also confirms the uniform dispersion of the fillers in the polymer matrix without the formation of voids (Fig 4e and 4f). Therefore, based on the experimental results and the fitting curves calculated by the Nielsen model shown in Fig 9, it may be concluded that appropriate filler modification and compatibilizer selection could greatly reduce the filler aggregation and improve the filler dispersion in the composites, which results in the retention of high aspect ratios of fillers and enhanced TCs of the composites.

## Conclusions

In this work, a bio-inspired polydopamine layers were coated onto BN surfaces, and the strong π-π interactions between polydopamine and BN allowed the non-covalent functionalization of BN, as confirmed by the SEM, TGA, FTIR and contact angle analysis. The catechol and amine groups in polydopamine could form chemical bonding with the compatibilizer, maleic anhydride grafted PP (PP-g-ma), resulting in much improved interfacial adhesion between the filler and matrix in the PP/PP-g-ma/f-BN composites than that in the PP/BN composites. In comparison, without the addition of PP-g-ma, PP/f-BN composites exhibited even worse filler-matrix compatibility because of the mismatch between relatively hydrophilic f-BN and relatively hydrophobic PP.

At the same filler content, the composite TCs always followed the same order of PP/PP-g-ma/f-BN > PP/BN > PP/f-BN, which was closely related to the morphology of the composites. The PP/PP-g-ma/f-BN composites demonstrated the best filler dispersion and the least void formation, and thus the phonon scattering at the filler-matrix interface was suppressed the most among the three series of PP composites. While for PP/f-BN composites, there showed the most voids and the most severe filler aggregation, causing the strongest interruption of the thermal paths. Compared to that of neat PP, the incorporation of 25 wt% (12 vol%) f-BN alone showed an enhancement of only 1.95 times in composite TC, while there was a TC enhancement of 2.14 times for the PP/BN composites with the same filler content. With the addition of 2.5 wt% PP-g-ma, the PP/PP-g-ma/f-BN composites achieved much larger TC improvement, with an enhancement of 2.64 times at 25 wt% (12 vol%) filler loading. Taking into account the effective aspect ratio of fillers, the Nielsen model was found to fit quite well with the experimental TC results. In summary, the introduction of polydopamine-modified BN was able to significantly improve the thermal conduction in the PP composites with the aid of PP-g-ma, emphasizing the importance of appropriate filler modification and compatibilizer selection on the thermal properties of the composites.

## Supporting Information

S1 Fig Thermal conductivity of the four series of polymer composites as a function of filler loading. (DOCX)
