# Supplementary material for: Thermal Conductivity Performance of Polypropylene Composites Filled with Polydopamine-Functionalized Hexagonal Boron Nitride
Source: PLoS One. 2017 Jan 20;12(1):e0170523. doi: 10.1371/journal.pone.0170523 (PMC5249180; doi:10.1371/journal.pone.0170523)
Supplement: S1 Fig — (DOCX) [file pone.0170523.s001.docx]

Supporting Information

for

Thermal conductivity performance of polypropylene composites filled with polydopamine-functionalized hexagonal boron nitride

Lin Chen^1^, Hong-Fei Xu^1^, Shao-Jian He^2^, Yi-Hang Du^1^, Nan-Jie Yu^1^,

Xiao-Ze Du^1,^*, Jun Lin^2,^*, Sergei Nazarenko^3^

1 Key Laboratory of Condition Monitoring and Control for Power Plant Equipment of Ministry of Education, North China Electric Power University, Beijing 102206, China

2 School of Renewable Energy, North China Electric Power University, Beijing 102206, China

3 School of Polymers and High Performance Materials, The University of Southern Mississippi, Hattiesburg, MS 39406, USA

*Corresponding author 1: Xiao-Ze Du +86-10-61773923 duxz@ncepu.edu.cn

*Corresponding author 2: Jun Lin +86-10-61772185 jun.lin@ncepu.edu.cn

S1 Fig. Thermal conductivity of the four series of polymer composites as a function of filler loading.
